# Fabrication and Characterization of Flexible Medical-Grade TPU Filament for Fused Deposition Modeling 3DP Technology

**DOI:** 10.3390/polym10121304

**Published:** 2018-11-25

**Authors:** Agnieszka Haryńska, Iga Gubanska, Justyna Kucinska-Lipka, Helena Janik

**Affiliations:** Polymer Technology Department, Chemical Faculty, Gdansk University of Technology, Narutowicza Street 11/12, 80-232 Gdansk, Poland; agnieszka.harynska@pg.edu.pl (A.H.); igaguban@pg.edu.pl (I.G.); heljanik@pg.edu.pl (H.J.)

**Keywords:** medical-grade filament, thermoplastic polyurethane, fused deposition modeling, filament forming, 3D printing

## Abstract

The possibility of using additive manufacturing (AM) in the medicine area has created new opportunities in health care. This has contributed to a sharp increase in demand for 3D printers, their systems and materials that are adapted to strict medical requirements. We described herein a medical-grade thermoplastic polyurethane (S-TPU) which was developed and then formed into a filament for Fused Deposition Modeling (FDM) 3D printers during a melt-extrusion process. S-TPU consisting of aliphatic hexamethylene 1,6-diisocyanate (HDI), amorphous α,ω-dihydroxy(ethylene-butylene adipate) (PEBA) and 1,4 butandiol (BDO) as a chain extender, was synthesized without the use of a catalyst. The filament (F-TPU) properties were characterized by rheological, mechanical, physico-chemical and in vitro biological properties. The tests showed biocompatibility of the obtained filament as well as revealed no significant effect of the filament formation process on its properties. This study may contribute to expanding the range of medical-grade flexible filaments for standard low-budget FDM printers.

## 1. Introduction

Additive manufacturing (AM) technologies have become a very effective and powerful tool in the health care industry [1,2,3]. Three-dimensional printers (3DP) are no longer the only rapid prototyping devices. The practical applications of an AM technology in medicine are more and more frequent. The use of 3D printers in combination with tools, such as computer-aided design (CAD) and radiographic methods (CT scans, MRI or X-rays), allows for the production of customized implants [4,5,6], or precise anatomical models for surgical planning [7,8]. Medical products fabricated via 3DP may be divided into five broad categories, i.e., surgical training systems (artificial organs, anatomical models), patient-matched devices (implants and prosthesis), tissue engineering constructs (scaffolds), pharmaceutical systems (drug delivery), as well reproduced tissues and organs. However, these medical products are characterized by different requirements and properties which are associated with the selection of proper material and a 3D printing technology. For example, reproduced temporal bones for drilling surgery training as well as artificial organs for a teaching purpose or preoperative planning should primarily exhibit high dimensional accuracy and organoleptic properties, e.g., texture, tactile sensation and viscoelasticity, which must imitate their biological counterparts [8,9]. Thus, 3D printed medical devices provide enormous assistance for surgeons and medical students. Moreover, tissue scaffolds, which have to provide physical support for new growing tissues and promote tissue regeneration, primarily should be highly biocompatible and degradable within a given timeframe. They should also have a three-dimensional, highly porous structure, and exhibit appropriate mechanical properties similar to the regenerated tissues [10,11]. These strict medical requirements limit the possibility of using conventional 3D printing materials or systems. Therefore, there is a need to look for and develop more advanced solutions and materials. AM technologies differ in operational principles, used equipment and materials. It is worth mentioning that, according to ASTM F2792-10 [12], AM is the official term but 3D printing (3DP) is a common definition of the family of AM technologies. Several widely used 3D printing technologies in medicine are: selective laser sintering (SLS), PolyJet (PJ), stereolithography (SLA) and fused deposition modeling (FDM) [13]. Bioprinting is a separate branch of AM technologies designed to reproduce functional tissues/organs or create bioactive tissue scaffolds. In this process, biomaterials, different types of living cells/stem cells, nutrients, growths factors or photoinitiators are used, depending on the chosen 3D bioprinting technology. The main 3D bioprinting techniques are: inkjet-based (thermal or acoustic inducted deposition drop by drop materials known as “bioinks” [14]), laser-based (deposition of biomaterial droplets using high energy lasers e.g., laser-inducted forward transfer LIFT [15]), microextrusion-based (pneumatic or mechanical extrusion of the bioinks through a syringe with a piston [16]), cell electrospinning-based (deposition of liquid biomaterial in the form of droplets or fibers using a strong electric field [17]) or stereolithography-based (using the light-induced photo-polymerization process to create 3D bio-structures [18]). In References [14,15,16,17,18], the particular types of bioprinting were explained in more detail.

These innovative solutions, more sophisticated than FDM, require highly advanced machinery, equipment and materials, which are used by collaborating specialists in material engineering, biotechnology and surgery. FDM technology is one of the easiest to use and most cost-effective 3DP technologies, both in terms of purchase and service. According to Oskuti et. al, objects printed with the use of FDM are significantly less toxic than SLA-printed parts for living organisms [19]. FDM has already been successfully used for printing biomedical devices [20,21]. Additionally, a fast-growing open-source community provides access to expert solutions and knowledge in the use of FDM 3DPs, which further reduces costs and facilitates the use of these devices. FDM is based on the layered deposition of plasticized polymeric material on a movable platform. The polymeric material in the form of a filament is fed to the miniature temperature-controlled extruder, where the plasticization takes place. Filament is a thin wire with a strictly defined diameter. One of the filament formation methods is a melt extrusion process [22]. There is a variety of commercially available filament types that exhibit a wide range of properties, however, medical-grade filaments market is still developing. One of the few companies supplying certificated medical-grade filaments dedicated for FDM 3DP is Poly-Med (Anderson, SC, USA). Their series of filaments (Lactoprene^®^ 100M, Caproprene^®^ 100M, Max-Prene^®^ 955, Dioxaprene^®^ 100M) have been recently examined by Mohseni et al. [23] as potential materials for tissue engineering 3D constructs. The mentioned filaments are based on polylactide (PLA), polycaprolactone (PCL), poly(lactic-*co*-glicolic acid) (PLGA), and polydioxanone (PDO). Whereas, Bioflex^®^ supplied by FiloAlfa (Ozzero, Italy) is a highly durable and flexible filament, belonging to the group of so-called thermoplastic elastomers (TPE). Its medical properties are confirmed by USP Class VI and ISO 10993-4/5/10 (cytotoxicity, hemolysis, intracutaneous and injection tests). However, degradation studies performed by our team showed that this material is highly resistant to acidic and alkaline environments (supplementary data 1). This significantly limits its use, for example, in tissue engineering constructs in which the material should degrade and resorb proportionally to the rate of tissue growth [24]. Currently, the most commonly used “medical” filaments for FDM 3D printers are thermoplastic biopolymers based on crystalline PLA or PCL. They provide satisfactory properties required for medical devices and structures such as biodegradability, bioresorbability and adequate/suitable durability [25,26,27]. However, there is lack of filaments available on the market, which provides properties (texture, flexibility, tactile sensation) that allow for native tissues or organs to mimic. These are important requirements in case of appropriate surgical training systems production via 3DP technology for the surgeons and medical students [9]. Materials that may be alternative to medical filaments based on PCL and PLA are properly designed thermoplastic polyurethanes (TPUs). According to the literature, they exhibit biocompatibility and hemocompatibility [28]. Moreover, TPU degradation rate can be controlled as well [29]. Biostable TPUs are widely known and used in medicine as prosthetics, implants, artificial blood vessels, or gene carriers [30]. What is more, the TPUs have been already successfully applied as tissue-engineered scaffold [31], nerve guidance channels [32], breast implants, dialysis membranes, or aortic grafts [33]. Additionally, we have not recorded certified medical-grade TPU filaments available on the market. Whereas, studies performed by Jung et al. [34] and Tsai et al. [35] confirm the legitimacy of using appropriately designed TPUs (based on aromatic polyether urethane and Tecoflex^®^, respectively) as filaments to fabricate advanced structures for the tissue engineering purpose via FDM. Looking for new flexible medical-grade materials for filament fabrication may contribute to the popularization of low-budget FDM printers as a cost-effective tool in health care.

Based on these premises, in this paper, we report our studies on novel flexible and medical-grade filament (F-TPU), dedicated for FDM 3D printing technology. For this purpose, we have synthesized TPU using raw materials suitable for the synthesis of biomedical polyurethanes [36] like aliphatic hexamethylene 1,6-diisocyanate (HDI), amorphous α,ω-dihydroxy(ethylene-butylene adipate) (PEBA) and 1,4 butandiol (BDO) as a chain extender. A discussable subject in terms of polyurethane synthesis is the application of organotin catalysts like dibutyltin dilaurate (DBTDL) and stannous octoate (Sn(Oct)_2_) [37,38]. To avoid the possible accumulation of these catalysts in the TPU matrix, which may affect the deterioration of biocompatibility and hemocompatibility of the material [39], we did not apply it in the synthesis of TPU described in this paper.

This study is divided into two main sections: The first section focuses on the synthesis in bulk and characterization of cast polyurethane (S-TPU) and filament fabrication (F-TPU) via melt-extrusion. The second part is devoted to impact assessment of the filament forming process on selected physico-chemical and in-vitro biological properties. S-TPU was processed in filament (1.75 mm diameter) via melt-extrusion process. Then, physico-mechanical, chemical and rheological properties (tensile test, hardness, FTIR, contact angle, melt flow rate (MFR)), were characterized. Finally, a series of preliminary biomedical studies, such as hemocompatibility and cytotoxicity tests (NIH 3T3 cells) were performed for both materials S-TPU and F-TPU in order to assess the influence of the processing procedure on their properties.

## 2. Experimental

### 2.1. S-TPU Synthesis

**Figure 1:**
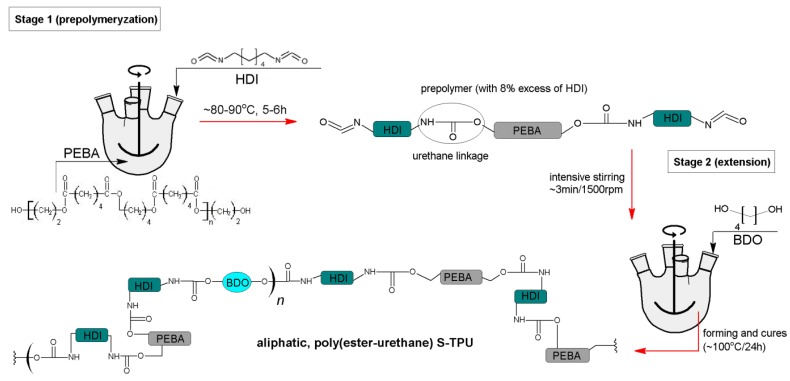
S-TPU synthesis scheme.

S-TPU was synthesized by standard two-step polymerization procedure (Figure 1) [28]. It was derived from amorphous PEBA (in contrary to polyurethanes (PURs) synthesized by using crystalline oligodiols [40,41]), aliphatic HDI and BDO chain extender. No catalysts were used in this synthesis (to avoid possible negative impact of the catalyst on the biocompatibility of the S-TPU [41,42]). In the first step, prepolymerization reaction (8 wt % of free isocyanate groups) was carried out at 90 °C for 6 h under vacuum by using PEBA and HDI. The reaction progress between PEBA and HDI over time was studied by the content of free isocyanate groups (F_NCO_ index) present in the prepolymer.

In the second step, the BDO was added to the reaction mixture in the molar ratio of unbounded isocyanate groups [NCO] (prepolymer) to hydroxyl groups [OH] (chain extender BDO) equal to 1.1:1. After 3 min of intensive stirring, the mixture was transferred into a mould, set at 80 °C, for 3 h. The gelling time of the reaction mixture in case of S-TPU obtained without the use of a catalyst was twice longer in comparison with TPU processed with 0.5% *w*/*w* of catalyst (dibutyltin dilaurate DBTDL) [36]. Finally, the samples were left in a heating furnace at 100 °C for 48 h to complete the reaction. At the end, obtained solid S-TPU was granulated to the size of 4 mm ± 1 mm (diameter) at room temperatures in a high-speed mill (speed 90 rpm, Wittmann Battenfeld, Grodzisk Mazowiecki, Poland) in order to have the material in the proper state be extruded. The detailed characteristics of used raw materials are given in Table 1.

### 2.2. Identification of Free Isocyanate Groups

The determination of free isocyanate groups (F_NCO_, %) was performed according to the PN-EN 1242:2006 standard. The percentage of free isocyanate groups was calculated by Formula (1);

(1) $$\;\%NCO=\;\frac{\left(V_{0}-V_{1}\right)*0.4}{m}\;$$

*% NCO*–percentage of unbound isocyanate groups (% mass)
*V*_0_—volume of HCl solution used for blank probe titration (cm^3^)
*V*_1_—volume of HCl solution used for the test sample titration (cm^3^)
*m*—sample mass (g]

### 2.3. Melt-Extrusion of F-TPU Filament

Double-screw extruder IQLINE (EHP 2 × 20 IQ, Zamak Mercator, Skawina, Poland) with nine heating zones, was used to obtain F-TPU filament. The custom-made molding nozzle diameter was equal to 1.5 mm and the L/D screw ratio was 22. Several parameter combinations were tested in order to obtain F-TPU filament of stable diameter dimension. Filament diameter was controlled by an electronic caliper.

### 2.4. Material Characterization Techniques

#### 2.4.1. Density

Six S-TPU samples of the 1 cm^2^ area were weighed with accuracy to 0.0001 g and then transferred to the analytical balance adjusted to density measurements (RADWAG AS 310/X, Radwag, Radom, Poland). Density was calculated in comparison to distilled water (1.0 g/cm^3^) at 20 °C.

#### 2.4.2. Melt Flow Rate (MFR)

S-TPUs MFR determination was carried out by using a load plastometer (ZWICK/Roell, Wrocław, Poland) according to the PN-EN ISO 1133-1:2011 standard. The value of MFR is expressed as a 1 g of material extruded through the standard capillary (2075 mm diameter) placed in a heating nozzle during 10 min (g/10 min). The S-TPU granules used in this study weighted 5 g/measurement. The conditions to perform MFR study for S-TPUs were as follows: 180 °C and 5 kg. Three repetitions were performed and the results were an average.

#### 2.4.3. Mechanical Characterization

Tensile strength and elongation at break were studied by using the universal testing machine Zwick & Roell Z020 (Wrocław, Poland) according to PN-EN ISO 527-2:2012 with a crosshead speed of 500 mm/min and initial force of 1 N. Five samples were studied and the results are an average. Hardness was measured by using Shore method according to PN-EN ISO 868:2005 standard. Obtained data were presented with Shore D degree (°Sh D). The results were an average of 10 measurements.

#### 2.4.4. Fourier Transform Infrared Spectroscopy (FTIR)

The FTIR analysis was performed with the use of Nicolet 8700 Spectrometer (Thermo Fisher Scientific, Waltham, MA, USA) in the spectral range from 4000 to 500 cm^−1^ averaging 256 scans with a resolution of 4 cm^−1^. The measurement was carried out both for the synthesized S-TPU and filament F-TPU.

#### 2.4.5. Optical Microscopy (OM)

The surface of solid S-TPU and filament F-TPU was studied via reflection microscope. Samples were gold coated in the sputter coater Quorum 150T E. OM (Quorum Technologies Ltd., Laughton, GB) was performed at ×300 magnification.

#### 2.4.6. Contact Angle (CA)

The CA of the solid S-TPU and filament F-TPU surfaces were determined at room temperature by using a Kruss Goniometer G10 (KRÜSS GmbH, Hamburg, Germany) with drop shape analysis software. A droplet of 2 µL volume was deposited on the samples surfaces and images were taken at the static conditions using a video instrument, drop shape analysis software DSA4. The results are an average of five measurement points randomly selected at the samples’ surface.

### 2.5. Biological Characterization

#### 2.5.1. Short-Term Hemocompatibility Test

Hemocompatibility test was conducted in Medical Academy Clinical Centre in Gdansk by using SYSMEX XS–1000i analyzer (Sysmex, Warszawa, Poland), according to the Polish ISO standard (PN-EN ISO 15189). Venous blood, from a healthy women served as a sample, which was placed in a sterile test-tube with an antithrombotic agent (potassium acetate), immediately after sampling. Then, the blood morphology of pure blood was conducted (served as reference parameters). Later, S-TPU and F-TPU samples were cut (8 cm^2^ surface) and immersed in 8 mL of blood and then, placed in the sterile test-tubes. All the test specimens (S-TPU and F-TPU) were previously sterilized by using argon plasma generated over H_2_O_2_. The incubation time of the samples (S-TPU and F-TPU) in blood was 15 min (at room temperature). After this time, the samples were removed and the blood was tested again. The results are an average of six measurements.

#### 2.5.2. Indirect Cytotoxicity Test

Cell Culture Mouse embryonic fibroblast NIH 3T3 cells were cultured in High Glucose Dulbecco’s modified Eagle’s medium (DMEM HG, Sigma Aldrich, Poznan, Poland) supplemented with 10% fetal bovine serum (FBS) and antibiotics (100 µg/mL each of penicillin and streptomycin) at 37 °C in a humidified atmosphere containing 5% CO_2_. The effect of indirect MTT Proliferation Assay of S-TPU or F-TPU exposure on NIH 3T3 cell proliferation was determined by 3-(4,5-dimethylthiazol-2-yl)-2,5-diphenyl tetrazolium bromide (MTT) colorimetric assay using 100% concentrations of samples extract. Sterilization of each side of the samples were conducted in 70% ethanol (30 min) and then by UV (exposure for 1 h). Then, MPLT samples were placed in DMEM HG complemented with 10% FBS and penicillin/streptomycin (24 h, 37 °C). The extraction medium volume was equal to 100 mg/mL. Extraction medium was subsequently filtered (0.2-µM filter) and 100% extracts of S-TPU and F-TPU were obtained. The 24-well plates were used for NIH 3T3 cells (2 × 104) seeding (for 24 h). After this time, the medium was changed to S-TPU/F-TPU extract and the incubation process was carried out for the next 24, 48 and 72 h. A mixture of DMEM HG, FBS and antibiotics was used as a non-toxic control sample. Finally, after the addition of 200 µL of MTT solution (4 mg/mL), the process of cell incubation begun (3 h, 37 °C). After removing the culture medium, the formazan crystals were dissolved in organic solvent (DMSO, Sigma Aldrich, Darmstadt, Germany). Optical density of obtained solutions was measured by iMark Microplate Absorbance Reader (Bio-Rad, Warsaw, Poland) at 570 nm. Results were presented as the percentage of cells proliferating after extract exposure relative to control cells cultured in extract-free medium. Obtained data are a mean of two separate experiments wherein each treatment condition was repeated in two wells.

#### 2.5.3. Statistical Analysis

For each extract, two tests were conducted and the results of the two experiments were averaged. For this purpose, two tests were conducted (ANOVA and Bonferroni) using GraphPad Prism 6 software (San Diago, CA, USA). p values of less than 0.05 were marked as significant (* *p* < 0.05; ** *p* < 0.01; *** *p* < 0.001; **** *p* < 0.0001; ns non-significant).

#### 2.5.4. Analysis Cells Morphology

Cellular morphology was studied using Zeiss inverted microscope with AxioCam digital camera (Zeiss, Göttingen, Germany). The samples placed in the 24-well plate were examined directly under the microscope.

## 3. Results and Discussions

### 3.1. S-TPU Synthesis—Identification of Free Isocyanate Groups

**Figure 2:**
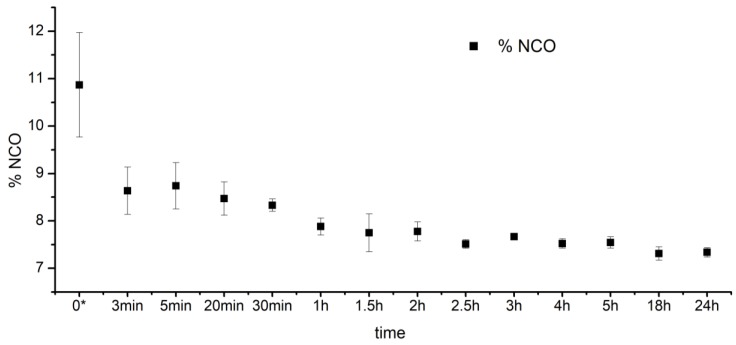
The changes of the isocyanate groups content (F_NCO_, %) over reaction time between PEBA and HDI (prepolymerization step); * time “0” is related to the moment when the PEBA and HDI were mixed together in a whole volume of the reactive mixture.

The reaction progress between PEBA and HDI over time is presented in Figure 2. After the first hour of the prepolymer synthesis, F_NCO_ index sharply decreased from 11% to ~8%. After the next 4–5 h, the F_NCO_ index stabilized at the level of ~8%, which indicated completion of the reaction between PEBA and HDI reagents. Thus, it can be concluded that PEBA and HDI react in a predictable and repeatable way, which is a significant aspect for further applications of these materials in the biomedical field.

### 3.2. Fabrication of F-TPU Filament from Synthesized S-TPU Granules

In Table 2, the selected melt-extrusion parameters used to fabricate the F-TPU filament, from S-TPU granules is presented. It can be seen that operating parameters are closely related to the temperature profile of S-TPU extrusion. During process 1 and 2 (Table 2), very high head pressure and machine load were noted. When the temperature profile was increased up to 210 °C, the head pressure and machine load dropped significantly, to about 17 bar and 15–18%, respectively. The further increase of the temperature profile (above 215 °C) caused a sharp decrease of head pressure (process 4, Table 2). This might be related to the viscosity of the melt polymer. High melt viscosity hinders the free flow of the polymer through the narrow forming die. Therefore, in process 1 and 2 (Table 2), the temperature profile was not high enough to ensure free flow of the polymer. Additionally, in the case of process 1 and 2, an enormous swelling of the polymer at the die exit (Barus effect) was observed. During process 4 (Table 2), the polymer underwent degradation.

In the process of a filament forming via melt-extrusion process, it is necessary to maintain a constant value of head pressure. In another way, it is not possible to obtain an extrudate with a stable dimension. The combination of the parameters in process 3* provided an appropriate profile which allowed us to obtain stable F-TPU filament with a constant diameter dimension (Table 2—highlighted).

### 3.3. Physico-Mechanical Properties of Synthesized S-TPU

The density of obtained S-TPUs were equal to 1.17 g/cm^3^, which is similar to the references, which reports typical PUR density in the range of 0.2–1.2 g/cm^3^ [43]. MFR is an important parameter of polymers processing, allowing for an assessment of using thermoplastic materials for further technological procedure. The MFR value is directly related to the melt viscosity at the test temperature as well with the test load. With the increase of sample viscosity, the flow rate decreases. Thermoplastic materials designed for injection molding are characterized by very high MFR value (very high flow-rate and low melt viscosity), in contrast to thermoplasts intended for extrusion. In the FDM process, the material in the form of a filament is plasticized in a mini-extruder and passes through a heated nozzle with a diameter in the range of 0.3–0.8 mm, and settles down at the movable platform. This is associated with a very short duration of heating and plasticizing of material. Therefore, the printed material should be relatively quick and easy to plasticize while maintaining the proper solidification rate, so that it will not flow from the layers built on the printer platform [44]. On the other hand, the strength and quality of the bonds formed between adjacent fibers depends on the growth of the neck formed among them and on the molar diffusion and randomization of the used polymeric filament across the interface [45,46]. Consequently, the degree of flow rate under FDM printing conditions should not be too high and sufficient/adequate for free flow of the filament out of the nozzle. Additionally, it should be added that higher MFR value allows for higher print speed. Thus, at a temperature of 200 °C and test load of 5 kg the MFR value of S-TPU was 40.74 ± 3.16 g/10 min, which might provide the free flow rate of the printed material.

Tensile strength of injection-molded S-TPU samples was 26 ± 2 MPa, which was close to values determined for commercially available medical-grade PURs, like Carbothane^®^ (39–67 MPa) and Desmopan^®^ (25–50 MPa) [47,48,49,50,51], as well in the range of elastic TPU filament NinjaFlex^®^ (26 MPa) [52]. Noted elongation at the break of obtained S-TPUs was of 706 ± 29% and higher than Tecoflex^®^ (365 ± 25%–400 ± 38% [53,54], the medical-grade PUR for biomedical applications and NinjaFlex^®^ filament (660%) [52]. Hardness of the obtained S-TPUs was 37.07 ± 0.80 °ShD and was comparable to the hardness of medical-grade TPU filament Bioflex^®^ (27 °ShD) [55]. It should be noted that as the filament hardness decreases, the difficulty of printing increases. This is particularly related to the folding of the filament on extruder rollers during the printing process. Mechanical properties of S-TPU correspond to those PURs obtained with the use of an organotin catalyst, dibutyltin dilaurate (DBTDL), described in our previous paper [56].

### 3.4. The Impact Assessment of Filament Formation on Selected S-TPU Properties

#### 3.4.1. Fourier Transform Infrared Spectroscopy (FTIR)

**Figure 3:**
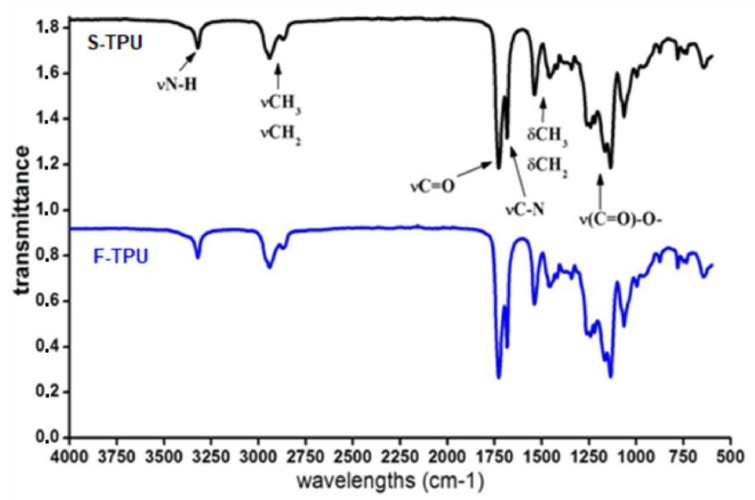
The FTIR spectra of S-TPU and extruded F-TPU filament.

FTIR spectra of obtained S-TPUs and extruded F-TPU filament was presented in Figure 3. The band assignments with a description were given in Table 3. The interpretation of the particular bands was made on the basis of a Silverstein et al. [57] scientific book. The presence of functional groups characteristic for poly(ester urethane)s was confirmed (Table 3) and the results are consistent with the interpretation given by Yiligor et al. [58]. The FTIR spectra of S-TPU and F-TPU are very similar, which might suggest that the extrusion process did not cause any chemical changes in S-TPU structure.

In both spectra, weak absorption peaks assigned to N–H stretching vibrations are observed at 3324 cm^−1^, which is related with the presence of hydrogen bonds between NH groups and macrodiol’s ester groups (C=O). The peaks, which appeared between 2941–2863 cm^−1^ correspond to the asymmetric and symmetric stretching vibrations of aliphatic CH_2_ groups presented in the S-TPU structure. Strong signals registered in the range of 1733–1685 cm^−1^ are related to stretching of C=O (both, hydrogen bonded and not hydrogen bonded in ester groups of macrodiol). Polyurethanes characteristic peak from C–N stretching are seen at 1535 cm^−1^. Peaks observed at 1465–1336 cm^−1^ correspond to deformation vibrations of aliphatic CH_2_ groups present in the S-TPU. Stretching vibrations of –NH–(C=O)–O– (urethane group), were registered at 1165 cm^−1^. In turn, stretching vibration of hydrogen bonded –C–(C=O)–O–, is presented between 1135–947 cm^−1^. Finally, peaks in the range of 873–863 are associated with out of plane bonding vibrations of C–H bending, CH_2_, NH, OH wagging and scissoring. According to physicochemical tables [57], absorbance in the range of 2250–2270 cm^−1^ is assigned to free NCO groups. The absence of those peaks indicates the complete reaction between reagents (HDI, PEBA, BDO) until the –NCO groups are completely converted into urethanes functional groups. This is also in accordance with the identification of free isocyanate groups during the pre-polymerization stage. FTIR analysis confirmed that F-TPU has the same chemical bonding type as bulk S-TPU and the filament formation process did not affect its chemical structure.

#### 3.4.2. Optical Microscopy (OM)

**Figure 4:**
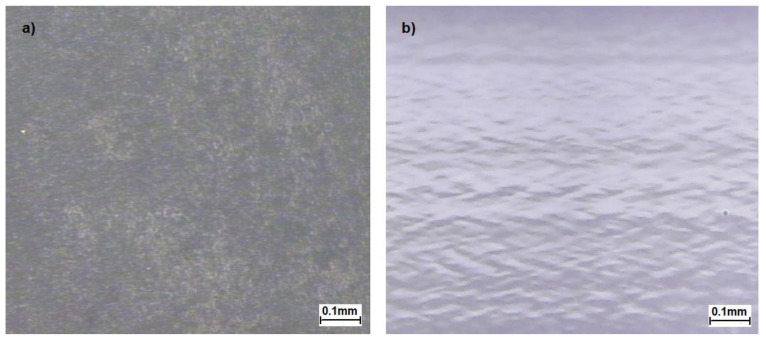
Optical microscopy of (**a**) bulk S-TPU and (**b**) F-TPU filament.

The optical microscopy of S-TPU and F-TPU filament was presented in Figure 4.

The surface of S-TPU (Figure 4a) is very rough and it does not reflect much of the light, so the particular patterns of the image are not clearly visible contrary to the image of extruded F-TPU (Figure 4b) surface. F-TPU surface is very smooth and very well reflects the light. A characteristic image of mound-depression nature for un-crosslinked TPUs [59] was observed (Figure 4b). The pattern in the image is oriented, which is obvious as the sample was extruded.

#### 3.4.3. Water Contact Angle (CA)

Water contact angle studies allow to specify the hydrophilicity/hydrophobicity of the material surface. However, CA is not a sufficient indicator to determine the biocompatibility of the material. Notwithstanding, hydrophilicity is an important biomedical parameter that favors the adherence and interaction of cells with material surfaces, thus CA studies provide preliminary biomedical characterization [60].

**Figure 5:**
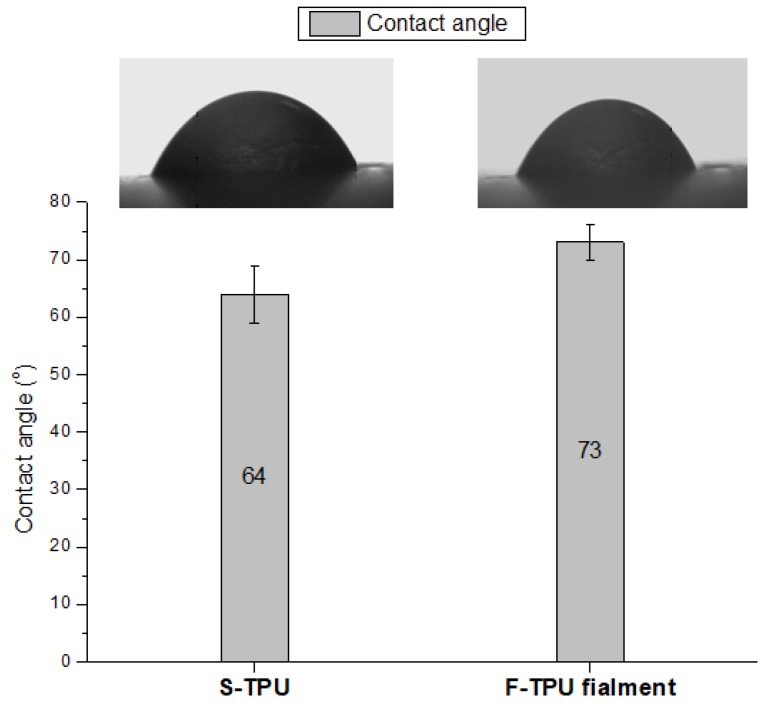
Contact angle of pure S-TPU and of extruded F-TPU filament.

Results of CA measurements of S-TPU and F-TPU were presented in Figure 5. The analysis of CA revealed that the extrusion process slightly increased the CA from 64° for S-TPU to 73° for F-TPU filament. Thus, the material became more hydrophobic after processing, which can be explained by the smoother surface presented by F-TPU filament. However, obtained values are still within the range of 55–75° that ensures proper adhesion of human cells to the surface of the selected material [50].

### 3.5. Biological Studies

#### 3.5.1. Short-Term Hemocompatibility Test

One of the test methods to evaluate biological properties in vitro is the study of blood response. Synthetic materials marked as medical-grade are intended for direct or indirect contact with body tissues, such as blood. Therefore, the study of the interaction of material with blood which is the fluid tissue present in every part of the body, seems to be important. It is a known fact that all of the biomaterials which are in contact with body tissues, cause the initiation of an inflammatory reaction (foreign body response FBR) [61]. Occurrence of acute or chronic reactions for a long time, disqualifies the material in medical applications. An initial interaction of cellular blood components with the artificial/synthetic surface occurs after the first few minutes of contact. Consequently, short-term studies of the interaction material—blood, can provide preliminary information about the biocompatibility of the material.

**Figure 6:**
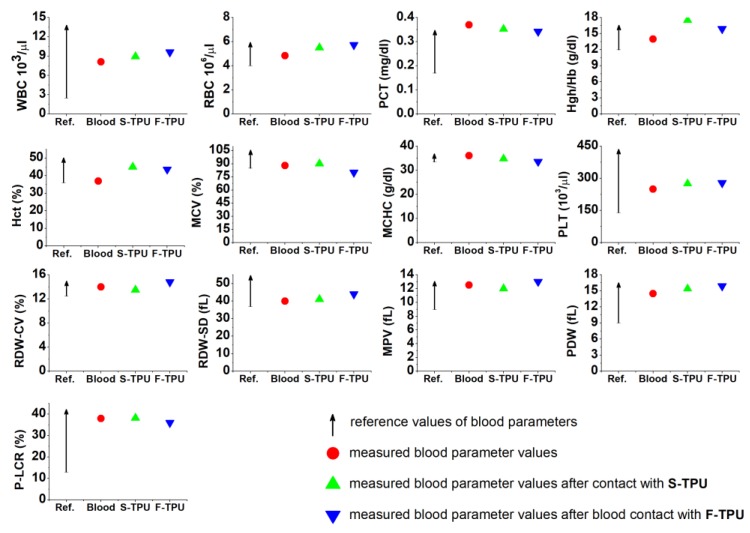
Short-term biocompatibility of S-TPU and extruded F-TPU filament with human blood. WBC—white blood cells (leucocytes); RBC—red blood cells (erythrocytes); PCT—percentage of platelets in whole blood volume; Hgh/Hb—hemoglobin; Hct—hematocrit; MCV mean corpuscular volume; MCHC—mean concentration of hemoglobin in blood cells; PLT—platelet amount (thrombocytes); RDW-CV/RDW-SD—distribution volume of red blood cells; MPV—mean platelet volume; PDW—indicator of platelet volume distribution; P-LCR—platelet larger cell ratio.

Analysis of the obtained data (Figure 6) showed that both synthesized S-TPU and extruded F-TPU filament can be pre-classified as biocompatible materials, under the specified conditions. Thus, the extrusion process did not influence this parameter and obtained materials may find a potential application in blood-contacting medical devices. This is consistent with the references related to the fact that PURs are one of the most hemocompatible synthetic polymers dedicated to medical applications [62].

It should be noted that all of the studied blood parameters are in the references range and they do not differ significantly from the values obtained for pure blood. Hemocompatibility test indicated that both S-TPU and F-TPU did not change the cell count of MCV, MCHC, PLT, RDW-CV, RDW-SD, MPV, PDW, and P-LCR. On the contrary to WBC, RBC, PCT, Hgh/Hb, Hct, which changed slightly. A slight decrease in the PCT value corresponding to the platelet count was observed. It can be related to aggregation and activation of platelets on the S-TPU surface [63]. This is an undesirable phenomenon that can lead to thrombosis [64]. Nevertheless, this value is still the norm. An increase of white blood cell (WBC) number was noticed, which is related to an initial inflammatory reaction that always takes place when in contact with an artificial organism. In turn, blood parameter associated with erythrocytes (RBC, Hgh/Hb, Hct) slightly increased to the maximum reference value, after contact with S-TPU and extruded F-TPU. Eventually, a significant reduction in RBC and Hct values could indicate the adhesion to the surfaces of erythrocytes, which in turn have a tendency to aggregate and form the so-called blood clots [65].

#### 3.5.2. Cytotoxicity

**Figure 7:**
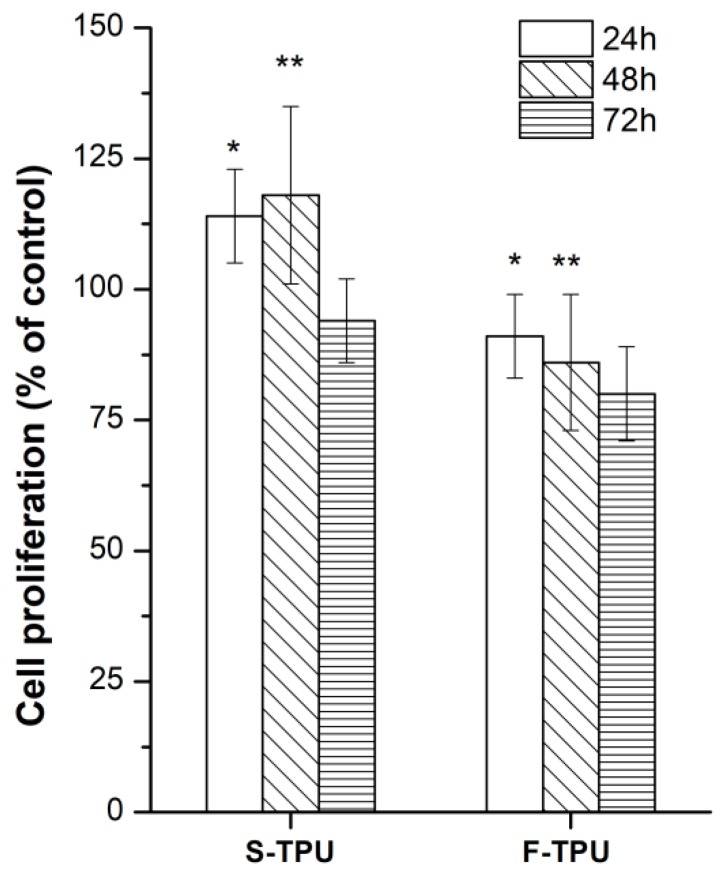
The effect of S-TPU and extruded F-TPU extracts on the in vitro growth of mouse embryonic fibroblast NIH 3T3 cells measured using MTT assay. Cell proliferation is represented as a percentage of control cell growth in cultures containing no S-TPU or extruded S-TPU filament extracts. Results are a mean ± SD of two separate experiments wherein each treatment condition was repeated in two wells. * *p* < 0.05; ** *p* < 0.001 vs. control.

The cytotoxicity of obtained S-TPU and F-TPU filament was shown in Figure 7. As it can be observed, both materials indicated biocompatibility towards NIH 3T3 cells. For S-TPU after 24 h and 48 h of incubation, the proliferation of cells was noted (over 100% of cells viability), while for F-TPU it was 95%, 86% and 79% after 24 h, 48 h, and 72 h, respectively. Slight differences in biocompatibility can be directly related to the higher hydrophilicity of the S-TPU surface than the extruded F-TPU. Moreover, the greater roughness and the more irregular the surface, the better adhesion of cells to the substrate, was noted [66], hence the possible difference in cell proliferation in the differentiation to F-TPU filament.

**Figure 8:**
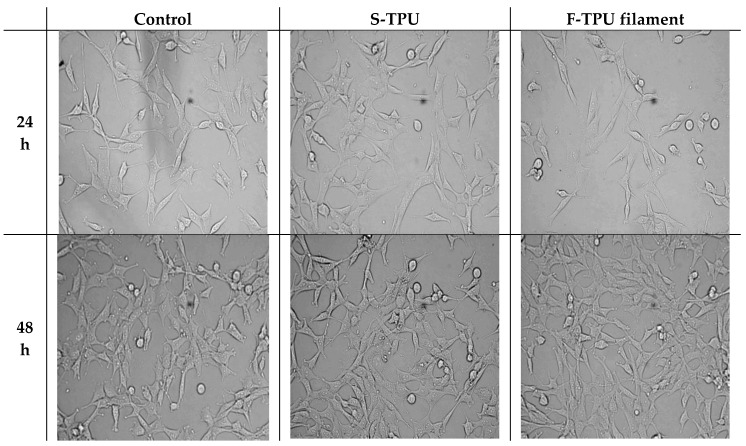

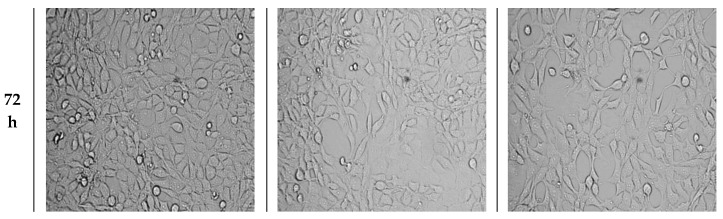
The effect of S-TPU and F-TPU filament extracts on the cellular morphology of mouse embryonic fibroblast NIH 3T3 cells.

Morphology of cells was observed for 72 h and the images are presented in Figure 8. As it can be seen, S-TPU and F-TPU filament extracts did not significantly change the morphology of the NIH 3T3 cells.

The morphology of NIH 3T3 cells did not change and was comparable to the control up to 72 h of incubation. Shape and cells dimensions were not impaired. It should be noted that there was a slight decrease in cell number when they had contact with the F-TPU filament, in comparison to the control sample. However, no cells degeneration or apoptosis was noticed during the incubation of both S-TPU and F-TPU. This might be explained by hindered cell adhesion to the F-TPU substrate, which exhibits a higher contact angle and smother surface than bulk S-TPU. Thus, these materials may be considered as suitable for biomedical applications.

## 4. Conclusions

In this work, we reported the synthesis, processing, physico-mechanical characterization and biological studies of new uncatalyzed aliphatic, amorphous polyurethane, as a potential medical-grade filament for using in FDM 3D printing technology.

For this purpose, bulk S-TPU with 1.1:1 NCO:OH molar ratio was synthesized and efforts have been made to adjust the temperature profile and operating parameters of S-TPU melt-extrusion. Established extrusion processing temperature for S-TPU was in the range of 160–205 °C, respectively. As a result, a stable F-TPU filament with 1.75 mm diameter was received. The mechanical characteristic and MFR of S-TPU is satisfactory, which has reference to FDM 3D printing, where the ease of processing, stability in print conditions and the proper flow, viscosity and hardness of the filament are responsible for the print quality. The summary of the obtained S-TPU mechanical properties and its comparison to the commercial medical-grade PURs in terms of mechanical efficiency is given in Table 4.

It can be seen that the mechanical properties of synthesized S-TPU are within the range of values suitable for medical-grade polyurethanes such as MillaMed^®^, Desmopan^®^ or Texin^®^. Thus, obtained uncatalysed aliphatic S-TPUs seems to be a promising candidate as a filament material for FDM 3D printer for medical purposes. Preliminary biological studies showed biocompatibility and hemocompatibility of F-TPU filament provided that this material may find application as a novel medical-grade, flexible filament for FDM 3DP. To confirm the validity of the presented studies, a test print of anatomical flexible heart using F-TPU filament and FDM type 3D printer, was made. Results are presented in supplementary data 2. The initial evaluation of FDM print with the use of obtained F-TPU filament allows to conclude that obtained F-TPU filament is suitable for 3D printing in the FDM type technology. Fabrication of F-TPU filament combined with 3DP technology allows for fabrication of customized and repeatable products without the use of toxic substances during printing. Moreover, the 3D printing technology in combination with elastomeric filament led to design cost-efficient and achievable patient-customized products.

## Figures and Tables

**Table 1:** Characteristic and chemical structure of used raw materials for the S-TPUs synthesis.

Compound	Supplier	Description	Structure Formula
BDO	Brenntag, Germany	Low molecular chain extender, Mol mass = 88 g/mol, Physical state–clear liquid, Purity > 95.5%, Tm = 204 °C, Boiling point ~ 230 °C, ρ (20 °C) = 1020 g/cm^3^	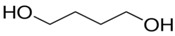
HDI	Sigma-Aldrich, Germany	Aliphatic diisocyanate, colorless liquid. Boiling point = 255 °C, Flash point = 130 °C, ρ (25 °C) = 1.05 g/cm^3^, Purity > 99%, Tm = −67 °C, Soluble in water, LD50 (rat) = 746 mg/kg.	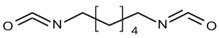
PEBA (POLIOS 55/20)	Purinova, Poland	Ester-based polyol, Mol mass = 2000 g/mol, Hydroxyl number = 54–58, Acid number–max. 0.6.	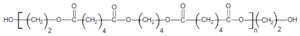

**Table 2:** Process parameters of F-TPU filament fabrication.

Lp.	Zones Temperature Profile [°C]											Operating Parameters		
	I	II	III	IV	V	VI	VII	VIII	IX	Coupler	Head	Rotation speed [rpm]	Head pressure [bar]	Load [%]
1	160	165	170	175	185	185	190	195	190	190	185	20	37–48	45–50
2	170	175	175	180	190	200	205	200	200	195	195	20	28–30	20–28
3 *	**170**	**175**	**180**	**190**	**200**	**205**	**210**	**210**	**205**	**200**	**200**	**20**	**17–18**	**15–18**
4	170	175	180	190	195	205	210	213	217	215	210	20	3–6	5–7

* Melt-extrusion profile that provide a dimensionally stable F-TPU filament.

**Table 3:** Band assignments noted at the FTIR spectra of S-TPU and F-TPU filament.

S-TPU	F-TPU	Band	Description
Wavelength (cm^−1^)			
3324 w	3324 w	νNH	Stretching of NH groups. These groups were hydrogen bonded with C=O of ester groups present in macrodiol.
2941 w, 2863 w	2939 w, 2865 w	νCH_2_, νCH_3_	Stretching of aliphatic asymmetric and symmetric CH_2 _groups present in the S-TPU chain and in the S-TPU filament
1730 vs. −1686 s	1733 vs. −1685 s	νC=O	stretching of C=O in ester groups of macrodiol,(hydrogen bonded and not hydrogen bonded)
1535 s	1535 s	νC–N	Stretching of C–N in urethane group
1459 w–1336 vw	1465 w–1346 w	δCH_2_	deformation vibrations of aliphatic CH_2_ groups present in the S-TPU and S-TPU filament: bending, wagging, scissoring in plane
1259 m–1219 m	1257 s–1216 m	νC–(C=O)–O	Stretching vibrations of –C–(C=O)–O– (ester group), not hydrogen bonded
1165 s	1165 m	νNH–(C=O)–O	Stretching vibrations of –NH–(C=O)–O– of urethane group
1129 s–994 w	1135 s–947 m	νC–(C=O)–O νC–O	Stretching vibration of hydrogen bonded –C–(C=O)–O–,
873 w–642 w	873 w–638 m	δCH_2_, δNH, δOH	out of the plane deformation of CH_2_(scissoring/wagging) as well as NH and OH groups (scissoring and wagging).

w (wagging), v(vibrating), s (scissoring).

**Table 4:** Comparison of available medical-grade polyurethanes properties with the synthesized uncatalyzed S-TPU system. (The data were taken from the material safety data sheets available on the manufacturers’ websites).

Value Range	MilaMed^®^	Desmopan^®^ AU	Texin^®^RxT50	S-TPU
TSb [MPa]	15–30	25–50	25–52	26
Eb [%]	540–565	470–880	320–770	705
HS [°Sh A/D]	no data found	60A–75D	70A–65D	26D
Chemical composition	Aliphatic polyether	Aromatic polyester	Aromatic polyether	Aliphatic polyester

## Supplementary Materials

The following are available online at http://www.mdpi.com/2073-4360/10/12/1304/s1. Supplementary data 1 provides the results and methodology of accelerated degradation studies on Bioflex^®^ and F-TPU filaments as well microscopic analysis of degraded samples. Supplementary data 2 presents trial of FDM 3D printing process using obtained F-TPU filament.

## References

[1] Javaid M., Haleem A. (2017). Additive manufacturing applications in medical cases: A literature based review. Alexandria J. Med..

[2] Schubert C., Van Langeveld M.C., Donoso L.A. (2014). Innovations in 3D printing: A 3D overview from optics to organs. Br. J. Ophthalmol..

[3] Ventola C.L. (2014). Medical Applications for 3D Printing: Current and Projected Uses. Pharm. Ther..

[4] Klammert U., Gbureck U., Vorndran E., Rödiger J., Meyer-Marcotty P., Kübler A.C. (2010). 3D powder printed calcium phosphate implants for reconstruction of cranial and maxillofacial defects. J. Cranio-Maxillofacial Surg..

[5] Bergmann C., Lindner M., Zhang W., Koczur K., Kirsten A., Telle R., Fischer H. (2010). 3D printing of bone substitute implants using calcium phosphate and bioactive glasses. J. Eur. Ceram. Soc..

[6] Lee M.-Y., Chang C.-C., Ku Y.C. (2008). New layer-based imaging and rapid prototyping techniques for computer-aided design and manufacture of custom dental restoration. J. Med. Eng. Technol..

[7] O’reilly M.K., Reese S., Herlihy T., Geoghegan T., Cantwell C.P., Feeney R.N.M., Jones J.F.X. Fabrication and Assessment of 3D Printed Anatomical Models of the Lower Limb for Anatomical Teaching and Femoral Vessel Access Training in Medicine. Anat. Sci. Educ..

[8] Qiu K., Zhao Z., Haghiashtiani G., Guo S.-Z., He M., Su R., Zhu Z., Bhuiyan D.B., Murugan P., Meng F. (2017). 3D Printed Organ Models with Physical Properties of Tissue and Integrated Sensors. Adv. Mater. Technol..

[9] Garcia J., Yang Z., Mongrain R., Leask R.L., Lachapelle K. (2017). 3D printing materials and their use in medical education: a review of current technology and trends for the future. BMJ Simul. Technol. Enhanc. Learn..

[10] Ikada Y. (2006). Challenges in tissue engineering. J. R. Soc. Interface.

[11] Hutmacher D.W. (2000). Scaffolds in tissue engineering bone and cartilage. Biomaterials.

[12] (2010). ASTM F2792-10, Standard Terminology for Additive Manufacturing Technologies.

[13] Kim G.B., Lee S., Kim H., Yang D.H., Kim Y.-H., Kyung Y.S., Kim C.-S., Choi S.H., Kim B.J., Ha H. (2016). Three-Dimensional Printing: Basic Principles and Applications in Medicine and Radiology. Korean J. Radiol..

[14] Cui X., Boland T. (2009). Human microvasculature fabrication using thermal inkjet printing technology. Biomaterials.

[15] Gaebel R., Ma N., Liu J., Guan J., Koch L., Klopsch C., Gruene M., Toelk A., Wang W., Mark P. (2011). Patterning human stem cells and endothelial cells with laser printing for cardiac regeneration. Biomaterials.

[16] Merceron T.K., Burt M., Seol Y.J., Kang H.W., Lee S.J., Yoo J.J., Atala A. (2015). A 3D bioprinted complex structure for engineering the muscle-tendon unit. Biofabrication.

[17] Palareti G., Legnani C., Cosmi B., Antonucci E., Erba N., Poli D., Testa S., Tosetto A. (2016). Comparison between different D-Dimer cutoff values to assess the individual risk of recurrent venous thromboembolism: analysis of results obtained in the DULCIS study. Int. J. Lab. Hematol..

[18] Miri A.K., Nieto D., Iglesias L., Goodarzi Hosseinabadi H., Maharjan S., Ruiz-Esparza G.U., Khoshakhlagh P., Manbachi A., Dokmeci M.R., Chen S. (2018). Microfluidics-Enabled Multimaterial Maskless Stereolithographic Bioprinting. Adv. Mater..

[19] Oskui S.M., Diamante G., Liao C., Shi W., Gan J., Schlenk D., Grover W.H. (2016). Assessing and Reducing the Toxicity of 3D-Printed Parts. Environ. Sci. Technol. Lett..

[20] Xu N., Ye X., Wei D., Zhong J., Chen Y., Xu G., He D. (2014). 3D artificial bones for bone repair prepared by computed tomography-guided fused deposition modeling for bone repair. ACS Appl. Mater. Interfaces.

[21] Vargas-Alfredo N., Dorronsoro A., Cortajarena A.L., Rodríguez-Hernández J. (2017). Antimicrobial 3D Porous Scaffolds Prepared by Additive Manufacturing and Breath Figures. ACS Appl. Mater. Interfaces.

[22] Melocchi A., Parietti F., Maroni A., Foppoli A., Gazzaniga A., Zema L. (2016). Hot-melt extruded filaments based on pharmaceutical grade polymers for 3D printing by fused deposition modeling. Int. J. Pharm..

[23] Mohseni M., Hutmacher D.W., Castro N.J. (2018). Independent evaluation of medical-grade bioresorbable filaments for fused deposition modelling/fused filament fabrication of tissue engineered constructs. Polymers (Basel)..

[24] O’Brien F.J. (2011). Biomaterials & scaffolds for tissue engineering. Mater. Today.

[25] Patrício T., Domingos M., Gloria A., Bártolo P. (2013). Characterisation of PCL and PCL/PLA scaffolds for tissue engineering. Procedia CIRP.

[26] Zein I., Hutmacher D.W., Tan K.C., Teoh S.H. (2002). Fused deposition modeling of novel scaffold architectures for tissue engineering applications. Biomaterials.

[27] Hutmacher D.W., Schantz T., Zein I., Ng K.W., Teoh S.H., Tan K.C. (2001). Mechanical properties and cell cultural response of polycaprolactone scaffolds designed and fabricated via fused deposition modeling. J. Biomed. Mater. Res..

[28] Kucinska-Lipka J., Gubanska I., Strankowski M., Cieśliński H., Filipowicz N., Janik H. (2017). Synthesis and characterization of cycloaliphatic hydrophilic polyurethanes, modified with L-ascorbic acid, as materials for soft tissue regeneration. Mater. Sci. Eng. C.

[29] Cauich-rodríguez J.V., Chan-Chan L.H., Hernandez-Sánchez F., Cervantes-Uc J.M., Pignatello R. (2012). Degradation of Polyurethanes for Cardiovascular Applications. Advances in Biomaterials Science and Biomedical Applications.

[30] Davis F.J., Mitchell G.R. (2008). Polyurethane Based Materials with Applications in Medical Devices. Bio-Materials and Prototyping Applications in Medicine.

[31] Kucińska-Lipka J., Gubanska I., Pokrywczynska M., Ciesliński H., Filipowicz N., Drewa T., Janik H. (2017). Polyurethane porous scaffolds (PPS) for soft tissue regenerative medicine applications. Polym. Bull..

[32] Borkenhagen M., Stoll R.C., Neuenschwander P., Suter U.W., Aebischer P. (1998). In vivo performance of a new biodegradable polyester urethane system used as a nerve guidance channel. Biomaterials.

[33] Lamba N.M.K., Woodhouse K.A., Cooper S.L., Lelah M.D. (1998). Polyurethanes in biomedical applications.

[34] Jung S.Y., Lee S.J., Kim H.Y., Park H.S., Wang Z., Kim H.J., Yoo J.J., Chung S.M., Kim H.S. (2016). 3D printed polyurethane prosthesis for partial tracheal reconstruction: A pilot animal study. Biofabrication.

[35] Tsai K.J., Dixon S., Hale L.R., Darbyshire A., Martin D., de Mel A. (2017). Biomimetic heterogenous elastic tissue development. npj Regen. Med..

[36] Kucińska-Lipka J., Gubanska I., Skwarska A. (2017). Microporous Polyurethane Thin Layer as a Promising Scaffold for Tissue Engineering. Polymers (Basel)..

[37] Park H., Gong M.-S., Knowles J.C. (2013). Catalyst-free synthesis of high elongation degradable polyurethanes containing varying ratios of isosorbide and polycaprolactone: physical properties and biocompatibility. J. Mater. Sci. Mater. Med..

[38] Kim H.-J., Kang M.-S., Knowles J.C., Gong M.-S. (2014). Synthesis of highly elastic biocompatible polyurethanes based on bio-based isosorbide and poly(tetramethylene glycol) and their properties. J. Biomater. Appl..

[39] Tanzi M.C., Verderio P., Lampugnani M.G., Resnati M., Dejana E., Sturani E. (1994). Cytotoxicity of some catalysts commonly used in the synthesis of copolymers for biomedical use. J. Mater. Sci. Mater. Med..

[40] Hassan M., Mauritz K., Storey R., Wiggins J. (2006). Biodegradable Aliphatic Thermoplastic Polyurethane Based on Poly(e-caprolactone) and L-Lysine Diisocyanate. J. Polym. Sci. Part A Polym. Chem..

[41] Heijkants R.G.J.C., Van Calck R.V., Van Tienen T.G., De Groot J.H., Buma P., Pennings A.J., Veth R.P.H., Schouten A.J. (2005). Uncatalyzed synthesis, thermal and mechanical properties of polyurethanes based on poly(ε-caprolactone) and 1,4-butane diisocyanate with uniform hard segment. Biomaterials.

[42] Barrioni B.R., De Carvalho S.M., Oréfice R.L., De Oliveira A.A.R., Pereira M.D.M. (2015). Synthesis and characterization of biodegradable polyurethane films based on HDI with hydrolyzable crosslinked bonds and a homogeneous structure for biomedical applications. Mater. Sci. Eng. C.

[43] Janik H.Z. (2005). Struktury nadcząsteczkowe i wybrane właściwości rozgałęzionych i usieciowanych poli(estro-uretanów), poli(etero-uretanów) i poli(uretano-biuretów) formowanych reaktywnie. Zesz. Nauk. Politech. Gdańskiej. Chem..

[44] Pietrzak K., Isreb A., Alhnan M.A. (2015). A flexible-dose dispenser for immediate and extended release 3D printed tablets. Eur. J. Pharm. Biopharm..

[45] Sun Q., Rizvi G.M., Bellehumeur C.T., Gu P. (2008). Effect of processing conditions on the bonding quality of FDM polymer filaments. Rapid Prototyp. J..

[46] Gkartzou E., Koumoulos E.P., Charitidis C.A. (2017). Production and 3D printing processing of bio-based thermoplastic filament. Manuf. Rev..

[47] Nezarati R.M., Eifert M.B., Dempsey D.K., Cosgriff-Hernandez E. (2015). Electrospun vascular grafts with improved compliance matching to native vessels. J. Biomed. Mater. Res. Part B Appl. Biomater..

[48] Qin Y., Liu R., Zhao Y., Hu Z., Li X. (2016). Preparation of Dipyridamole/Polyurethane Core–Shell Nanofibers by Coaxial Electrospinning for Controlled-Release Antiplatelet Application. J. Nanosci. Nanotechnol..

[49] Wang H., Feng Y., Fang Z., Yuan W., Khan M. (2012). Co-electrospun blends of PU and PEG as potential biocompatible scaffolds for small-diameter vascular tissue engineering. Mater. Sci. Eng. C.

[50] Wang H., Feng Y., An B., Zhang W., Sun M., Fang Z., Yuan W., Khan M. (2012). Fabrication of PU/PEGMA crosslinked hybrid scaffolds by in situ UV photopolymerization favoring human endothelial cells growth for vascular tissue engineering. J. Mater. Sci. Mater. Med..

[51] Yuan W., Feng Y., Wang H., Yang D., An B., Zhang W., Khan M., Guo J. (2013). Hemocompatible surface of electrospun nanofibrous scaffolds by ATRP modification. Mater. Sci. Eng. C.

[52] NinjaTek Technical Sepcification of NinjaFlex 3D Printing Filament. https://ninjatek.com/wp-content/uploads/2016/05/NinjaFlex-TDS.pdf.

[53] Chen R., Huang C., Ke Q., He C., Wang H., Mo X. (2010). Preparation and characterization of coaxial electrospun thermoplastic polyurethane/collagen compound nanofibers for tissue engineering applications. Colloids Surfaces B Biointerfaces.

[54] Detta N., Errico C., Dinucci D., Puppi D., Clarke D.A., Reilly G.C., Chiellini F. (2010). Novel electrospun polyurethane/gelatin composite meshes for vascular grafts. J. Mater. Sci. Mater. Med..

[55] Filoalfa–Bioflex Filament. https://www.filoalfa3d.com/en/filaments-175mm/296-bioflex-pla-shore-27d-white-o-175-mm-8050327032385.html.

[56] Kucinska-Lipka J., Gubanska I., Sienkiewicz M. (2017). Thermal and mechanical properties of polyurethanes modified with L-ascorbic acid. J. Therm. Anal. Calorim..

[57] Socrates G. (2004). Infrared and Raman Characteristic Group Frequencies: Tables and Charts.

[58] Yilgor I., Yilgor E., Guler I.G., Ward T.C., Wilkes G.L. (2006). FTIR investigation of the influence of diisocyanate symmetry on the morphology development in model segmented polyurethanes. Polymer (Guildf)..

[59] Janik H. (2010). Progress in the studies of the supermolecular structure of segmented polyurethanes. Polimery.

[60] Menzies K.L., Jones L. (2010). The impact of contact angle on the biocompatibility of biomaterials. Optom. Vis. Sci..

[61] Anderson J., Rodrigues A., Chang D. (2008). Ferogin Body Reaction To Biometerials. Semin. Immunol..

[62] Fromstein J.D., Woodhouse K.A. (2002). Elastomeric biodegradable polyurethane blends for soft tissue applications. J. Biomater. Sci. Polym. Ed..

[63] Xin Z., Du B., Wang Y., Qian S., Li W., Gao Y., Sun M., Luan S., Yin J. (2017). Hemocompatibility Evaluation of Polyurethane Film with Surface-Grafted Sugar- Based Amphipathic Compounds. J. Anal. Bioanal. Tech..

[64] Williams D.F. (2008). On the mechanisms of biocompatibility. Biomaterials.

[65] Tahara D., Oikawa N., Kurita R. (2016). Mobility enhancement of red blood cells with biopolymers. J. Phys. Soc. Japan.

[66] Keshel S.H., Azhdadi S.N.K., Asefnejad A., Sadraeian M., Montazeri M., Biazar E. (2011). The relationship between cellular adhesion and surface roughness for polyurethane modified by microwave plasma radiation. Int. J. Nanomedicine.

