# Supplementary material for: Fabrication and Characterization of Flexible Medical-Grade TPU Filament for Fused Deposition Modeling 3DP Technology
Source: Polymers (Basel). 2018 Nov 25;10(12):1304. doi: 10.3390/polym10121304 (PMC6401970; doi:10.3390/polym10121304)
Supplement: Supplementary file 1 [file polymers-10-01304-s001.pdf]

Supplementary materials

Supplementary data 1

Degradation study

Accelerated degradation was performed for filaments Bioflex® (Filoalfa, Italy) and F-TPU, using 5M KOH and 2M HCl media. Cut samples (1,5 cm length) were immersed in the respective dilutions and incubated at 37°C for 30 days. At each respective time point (7, 14, 2 and 30 days), degradated samples were carefully rinsed out with deionizer water and dried in laboratory oven at 40°C for 48h. Mass loss was calculated as following;

$$Ms\ (\%) = \frac{m_0 - m_1}{m_0} * 100\ \% \qquad (1)$$

Where, (m_0) is initial mass of sample and (m_1) is residual mass.

The test result is shown in the graph below.

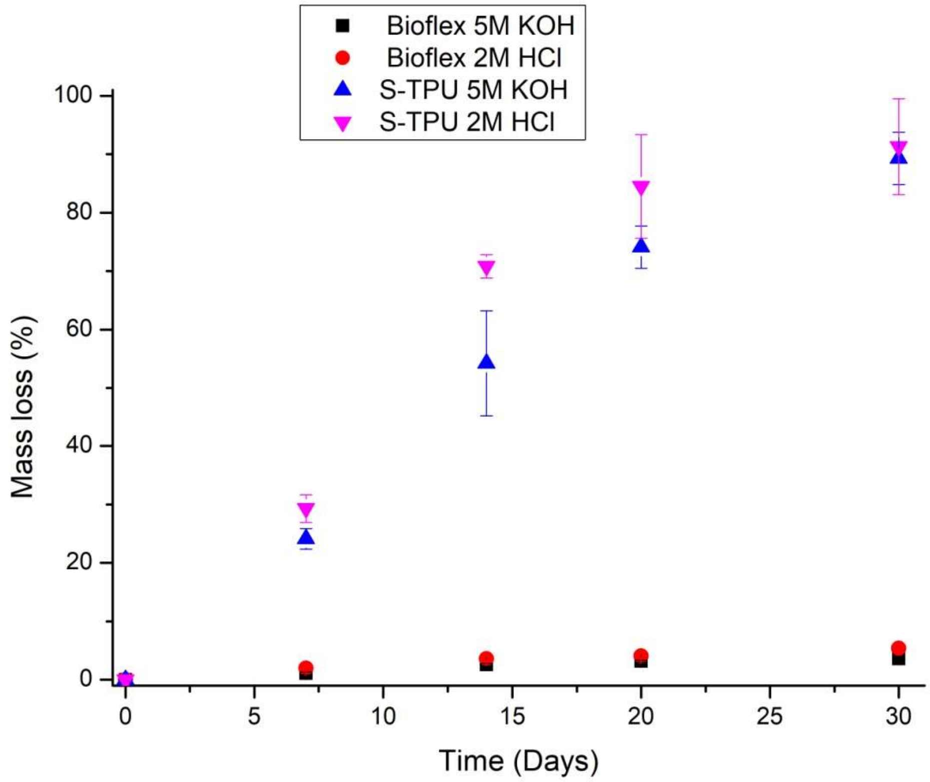

Figure S1 Accelerated degradation of Bioflex® and F-TPU filaments in 5M KOH and 2M HCl medium. Results are represented as mean ± SD (n = 4)

Optical microscopy

The surfaces of pristine and degraded filaments were analyzed by using optical microscope (OM) Delta Optical Genetic Pro (at x80 magnification).

Table S1 OM images of pristine and degradated filaments in KOH medium (x80).

Sample	0 days	7 days	20 days
Bioflex®	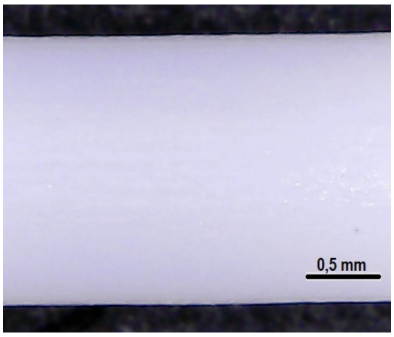	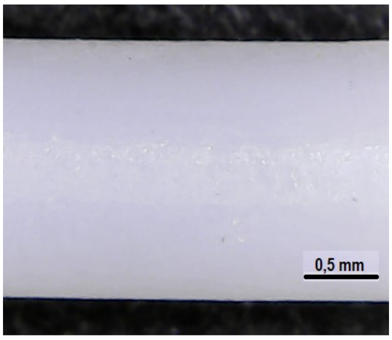	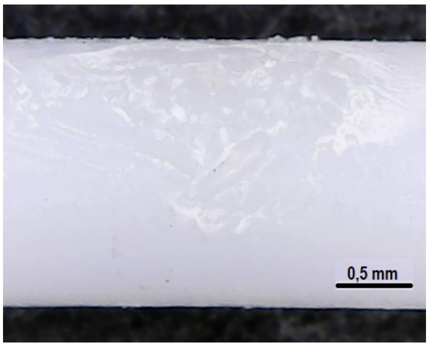
F-TPU	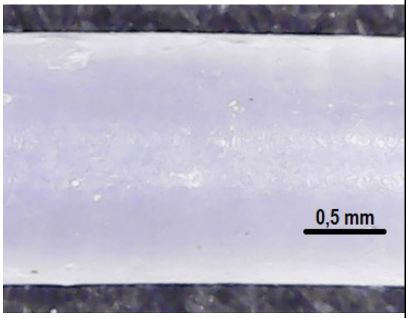	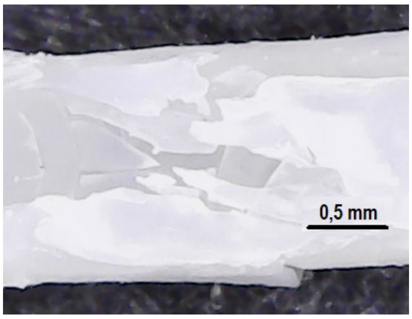	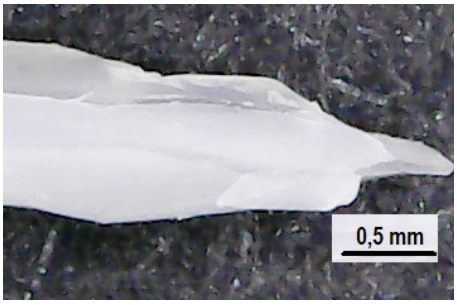

Table S2 OM images of pristine and degradated filaments in HCl medium (x80).

Sample	0 days	7 days	20 days
Bioflex®	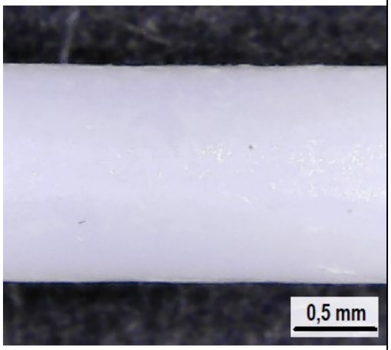	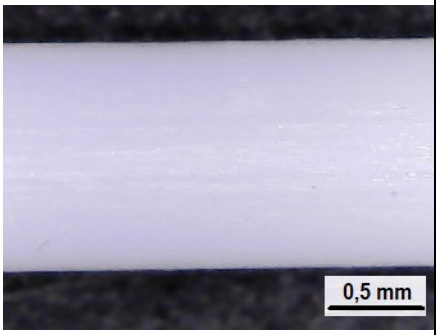	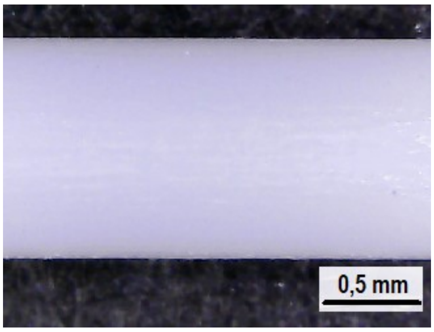

F-TPU	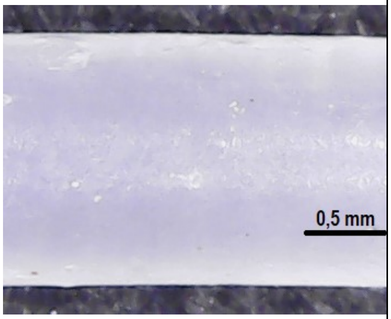	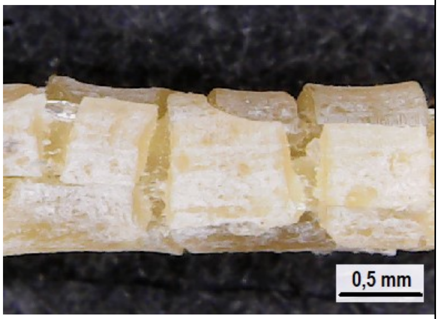	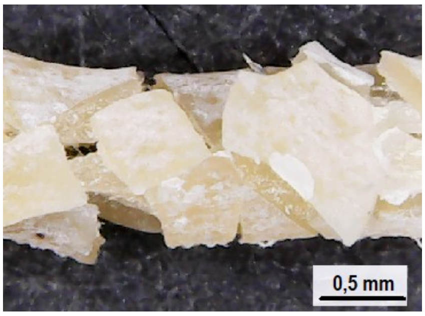

Table S3 Comparison of Bioflex and F-TPU after 20 days of incubation in KOH and HCl medium (x80)

20 days in KOH	20days in HCl
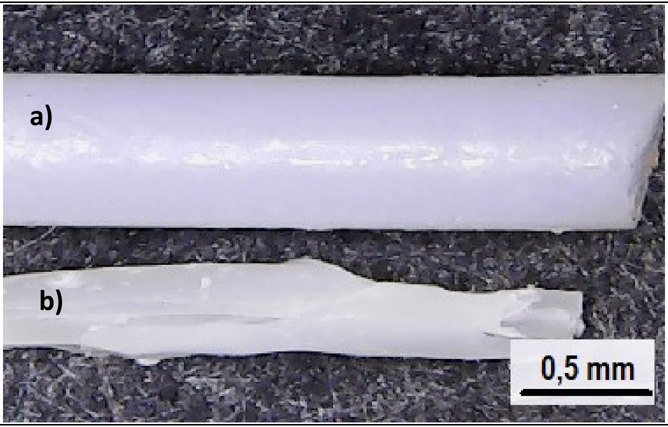	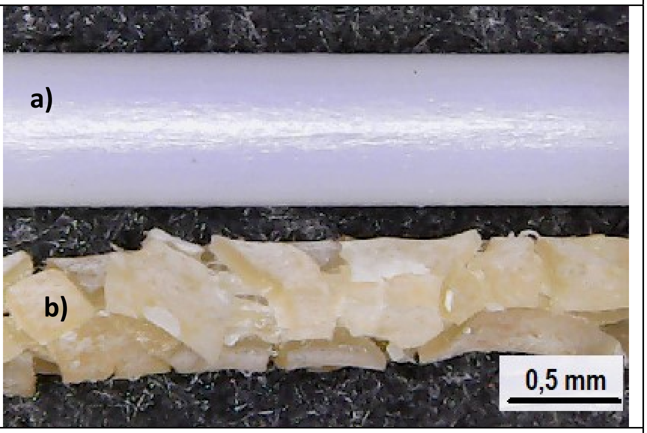
a) Bioflex<br>b) F-TPU	

Supplementary data 2

Initial FDM 3D printing of F-TPU filament

For preliminary evaluation of F-TPU filament potential use in FDM 3D printers, the test of printing was performed. For this purpose, we used ready-made SLT. format file of anatomical heart, available on 3dprint.nih.gov website (NIH 3D Print Exchange – an open-source community). This STL. file was converted into the printer control code "g-code" using an open-source program (Slic3r 1.2.9). The model was printed using single-head FDM-type 3D printer (self-made printer, Gdansk, Poland) (Figure S2.). The proper parameters of printing were as follow; printing speed, 25mm/s; printing temperature, 205-210°C; bed temperature, 55°C, fill density, 80%; layer thickness, 0.4mm. The printing time was about 8 hours.

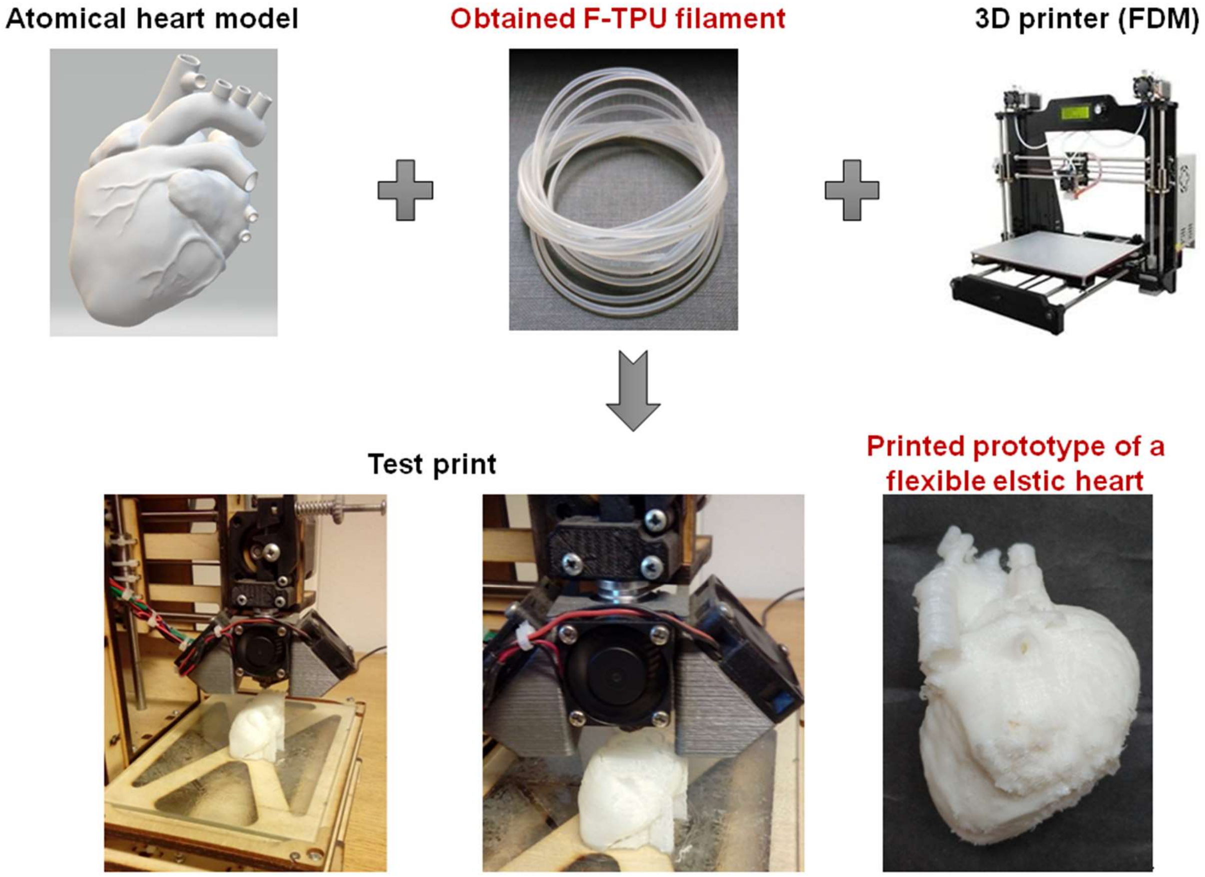

Figure S2 Scheme of the initial FDM 3D printing of heart by using F-TPU.

The initial evaluation of FDM print with the use of obtained F-TPU filament allows to conclude that obtained F-TPU filament is suitable for 3D printing in the FDM type technology.
